# Supplementary figures and images for: Temporal dynamics of animacy categorization in the brain of patients with mild cognitive impairment
Source: PLoS One. 2022 Feb 23;17(2):e0264058. doi: 10.1371/journal.pone.0264058 (PMC8865635; doi:10.1371/journal.pone.0264058)

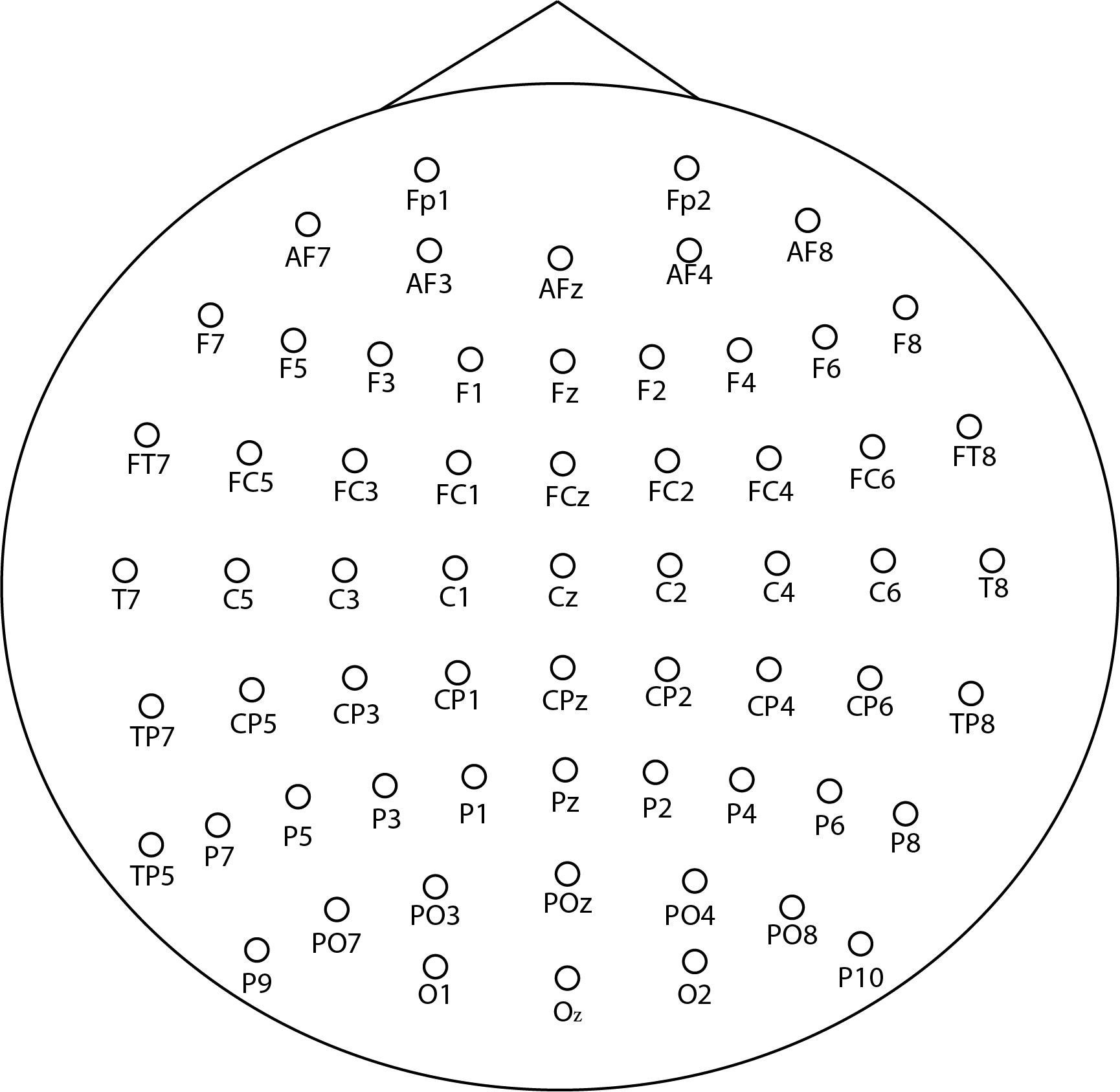

Supplement: S1 Fig — We used a 64-channel g.tec product at a sampling rate of 1200 Hz for EEG data acquisition (S1 Fig). The reference electrode (#33) was placed on the participant’s right ear. Fp: prefrontal; AFL: left anterior frontal; AFR: right anterior frontal; FL left frontal; Fz: midline frontal; FR: right frontal; FCL: left fronto-central; FCR: right fronto-central; TL: left temporal; CL: left central; Cz: midline central; CR: right central; TR: right temporal; TPL: left temporo-parietal; CPL: left centro-parietal; CPR: right centro-parietal; TPR: right temporo-parietal; PL: left parietal; PR: right parietal; POL: left parieto-occipital; Pz: midline parietal; POR: right parieto-occipital; O: occipital. (TIF) [file pone.0264058.s001.tif]
