# Supplementary material for: Temporal dynamics of animacy categorization in the brain of patients with mild cognitive impairment
Source: PLoS One. 2022 Feb 23;17(2):e0264058. doi: 10.1371/journal.pone.0264058 (PMC8865635; doi:10.1371/journal.pone.0264058)
Supplement: S1 Table — The absent subjects were removed from this study either due to their status of disease (AD or mild AD) or that their status changed during the development of the study. (PDF) [file pone.0264058.s002.pdf]

Subject	Label	MoCA	ACE-R	ICA Accuracy	ICA Speed	ICA Score
S01	HC	22	91	68	92	63
S02	MCI	25	90	74	76	56
S06	MCI	27	90	94	70	65
S07	MCI	27	93	76	85	65
S08	MCI	24	90	66	51	34
S09	MCI	24	93	90	76	68
S11	HC	24	85	84	82	69
S12	HC	25	92	82	83	68
S13	HC	28	98	92	82	76
S14	HC	28	97	90	79	71
S15	HC	28	95	61	85	52
S16	MCI	19	80	69	79	55
S17	MCI	25	90	79	72	57
S18	HC	25	95	89	85	75
S19	HC	27	95	93	77	72
S20	MCI	23	89	77	71	54
S21	HC	28	100	82	83	68
S25	MCI	28	88	84	68	57
S27	MCI	25	95	80	79	64
S28	HC	27	93	70	79	55
S29	HC	22	92	84	80	67
S30	HC	29	96	82	75	61
S31	MCI	26	92	82	79	65
S32	HC	24	82	83	93	77
S33	HC	27	97	85	77	66
S34	HC	29	91	92	72	66
S35	MCI	24	94	84	85	71
S36	HC	30	97	89	79	71
S38	HC	28	96	82	89	73
S39	HC	28	96	93	75	70
S40	MCI	20	85	60	66	39
S41	MCI	25	84	85	73	62
S42	MCI	25	82	87	67	59
S44	MCI	22	91	88	85	75

S45	MCI	26	94	81	88	72
S46	HC	25	92	83	83	69
S47	HC	27	93	93	87	81
S48	HC	23	95	91	81	74
S49	MCI	23	88	80	82	66
S52	HC	22	94	92	72	66
